# Region-specific neuroprotective effects of meldonium pretreatment in two models of sepsis-associated encephalopathy

**DOI:** 10.3389/fphar.2025.1588831

**Published:** 2025-04-30

**Authors:** Aleksandra Ružičić, Tijana Srdić, Katarina Bobić, Dunja Drakulić, Filip Veljković, Ana Todorović, Siniša Đurašević, Iva Lakić

**Affiliations:** ^1^ Department of Animal and Human Physiology, Institute for Physiology and Biochemistry Ivan Đaja, Faculty of Biology, University of Belgrade, Belgrade, Serbia; ^2^ Department of Molecular Biology and Endocrinology, VINČA Institute of Nuclear Sciences, National Institute of the Republic of Serbia, University of Belgrade, Belgrade, Serbia; ^3^ Department of Physical Chemistry, VINČA Institute of Nuclear Sciences, National Institute of the Republic of Serbia, University of Belgrade, Belgrade, Serbia

**Keywords:** sepsis, model, sepsis-associated encephalopathy (SAE), meldonium (mildronate), brain, rat

## Abstract

Sepsis-associated encephalopathy (SAE) is a common yet poorly understood complication of sepsis, which poses a burden in clinical settings, as its management relies on supportive care without targeted pharmacological interventions. Meldonium is a drug approved for ischemic heart disease but has also gained attention for its neuroprotective effects. In animal models of sepsis, meldonium pretreatment exerted antioxidative, antiapoptotic, and anti-inflammatory effects, but its neurological effects have not been studied in SAE. In the present study, rats were pretreated with meldonium for 4 weeks, before sepsis was induced via a faecal intraperitoneal injection (FIP) or a lipopolysaccharide (LPS) injection. The cerebellum, medulla oblongata, and prefrontal cortex were examined due to their involvement in functions that are often impaired in sepsis. Eight hours post-sepsis induction, markers of brain injury were assessed, including reflexes scores, dry to wet brain mass ratio, prooxidant-antioxidant balance (PAB), advanced oxidation protein products (AOPP), lipid peroxidation (LPO), phosphatidylcholine (PC) to lysophosphatidylcholine (LPC) ratio, HMGB1 and haptoglobin protein expression, and CD73 activity. Meldonium-pretreated FIP-septic rats showed an earlier decline in reflex scores compared to the sepsis-only group, accompanied by a slight brain water accumulation. However, in both models of sepsis, meldonium pretreatment prevented alterations in the PAB, AOPP, and LPO in a region-specific manner. It also preserved the PC/LPC ratio in the prefrontal cortex of FIP-septic rats and in all regions of LPS-septic rats. Haptoglobin protein content was altered only in FIP-septic rats, and preserved by meldonium pretreatment in the cerebellum and medulla oblongata of these rats. Additionally, meldonium pretreatment preserved CD73 activity in the medulla oblongata and prefrontal cortex of FIP-septic rats and in the cerebellum and prefrontal cortex of LPS-septic rats. In conclusion, our study is the first to demonstrate that pretreatment with meldonium, a drug that has shown neuroprotective effects in other invasive models can also provide benefits in SAE, with the extent of protection depending on both the model of sepsis induction and the specific brain region investigated. Our findings support the discussion on the importance of selecting the right sepsis model and studying individual brain regions when investigating SAE and potential therapeutic approaches.

## 1 Introduction

Sepsis is a life-threatening condition caused by an uncontrolled immune response to an infection, and if not recognized and treated in time, it inevitably leads to organ failure and death (Singer et al., 2016). One of the most common complications is sepsis-associated encephalopathy (SAE), which encompasses both short- and long-term brain damage through mechanisms not fully elucidated (Catarina et al., 2021; Sekino et al., 2022). Proposed mechanisms of SAE onset and progression include disruption of cerebrovascular components and the blood-brain barrier (BBB), neurotransmitter and mitochondrial dysfunction, systemic inflammation, neuroinflammation (Aslankoc et al., 2018; Olivieri et al., 2018; Tian et al., 2019), oxidative stress (Aslankoc et al., 2018; Pan et al., 2022), hypoperfusion (Pfister et al., 2008), accumulation of damaged proteins (Brown, 2019; Kirk et al., 2019), direct brain damage (Sekino et al., 2022), etc. Current therapy for sepsis-induced brain injury focuses solely on treating the symptoms, as there are no treatment options that specifically target the underlying mechanisms of brain dysfunction (Sekino et al., 2022).

Meldonium (3-(2,2,2-trimethylhydrazinium) propionate) is the active substance of Mildronate^®^ (Dambrova et al., 2002; Sesti et al., 2006), an orally administered prescription drug used in Eastern Europe for treating ischemic conditions of the heart and brain (Pupure et al., 2010a; Zhu et al., 2013; Kotov et al., 2015). When taken orally, meldonium is absorbed in the small intestine via active transport, enters the bloodstream where it binds to plasma lipids, and is transported into cells expressing the organic cation transporter 2 (OCTN2), which also facilitates its passage across the blood-brain barrier (Berlato and Bairros, 2020). Within the cell, meldonium inhibits the biosynthesis and transport of L-carnitine, thereby reducing the transport of free fatty acids (FAs) into the mitochondria and their subsequent oxidation (Dambrova et al., 2016). In this way, meldonium prevents the accumulation of harmful oxidative intermediates in the mitochondria (Dambrova et al., 2016). This is of particular interest in the context of sepsis, where mitochondrial damage due to excessive production of reactive oxygen species (ROS) leads to an increased energy demand (Zhang et al., 2018).

The neuroprotective properties of meldonium have been demonstrated in various experimental models (Sjakste et al., 2005; Pupure et al., 2010a; Pupure et al., 2010b; Zvejniece et al., 2010; Isajevs et al., 2011; Beitnere et al., 2014; Demir et al., 2019; Ozaydin et al., 2023). In the cortex of mice with azidothymidine-induced neurotoxicity, meldonium reduced infiltration of inflammatory cells and apoptosis (Pupure et al., 2010a). Similar observations were reported in the rat model of traumatic brain injury (Demir et al., 2019). In a model of peripheral nerve damage, meldonium halted neuronal degeneration, reduced neuroinflammation, and alleviated associated pain behavior (Pupure et al., 2010b). Ozaydin et al. recently reported meldonium’s protective effects in rabbits with spinal cord injury (Ozaydin et al., 2023). In both seizure and ethanol intoxication rat models, a single intraperitoneal injection of meldonium exerted anticonvulsant and antihypnotic effects (Zvejniece et al., 2010). Perhaps most interestingly, in a mouse model of Alzheimer’s disease, meldonium improved cognitive performance and reduced amyloid-β deposition in the hippocampus (Beitnere et al., 2014). In a rat model of Parkinson’s disease, meldonium preserved the expression of proteins crucial for the neuronal protection (Isajevs et al., 2011).

Even though neuroprotective effects of meldonium have been demonstrated in other invasive models, present study, to our knowledge, would be the first to explore its potential benefits in the context of SAE. This requires the investigation of different brain regions, as regional susceptibility has been reported in sepsis-induced brain injury (Sharshar et al., 2003; Kafa et al., 2010; Heming et al., 2017). The cerebellum, medulla oblongata, and prefrontal cortex were chosen due to their critical roles in motor control, autonomic function, and higher cognitive processes, all of which are significantly affected in sepsis (Heming et al., 2017; Zhao et al., 2024). To fill these gaps, the present study investigated the effects of meldonium pretreatment on these brain regions in two commonly used models of experimentally induced sepsis.

## 2 Materials and methods

### 2.1 Ethical approval

All animals were obtained from the animal facility of the Faculty of Biology, University of Belgrade, Republic of Serbia. The animals were group-housed two per cage and acclimated to standard conditions at a temperature of 22°C ± 1°C and a 12-h light-dark regime. Throughout the study, animals had *ad libitum* access to standard laboratory chow and water. Ethical approval for the experiments was granted by the Veterinary Directorate of the Serbian Ministry of Agriculture, Forestry and Water Management (permits No. 323-07-11574/2021-05 and 323-07-05650/2021-05/1). All procedures involving animals were performed in accordance with Serbian Animal Welfare Law, Directive 2010/63/EU, and the ARRIVE guidelines for reporting animal research. Given the invasiveness of the sepsis models, animal welfare was closely monitored and assessed based on behavior and general health status, with humane endpoints implemented accordingly.

### 2.2 Meldonium pretreatment

Analytical grade meldonium was purchased from Shenzhen Calson Bio-Tech Co. Ltd. (China). Meldonium was dissolved in tap water, and fresh meldonium solution was prepared three times a week. The rats had *ad libitum* access to either tap water or meldonium solution via standard plastic bottles placed on the cages. Both body mass and daily consumption of water/meldonium solution were monitored for 2 weeks before the experiment and throughout the 4-week meldonium treatment period. The concentration of the meldonium solution was adjusted to ensure a daily intake of 300 mg per kg of body mass. This dose, within the range of previously studied animal doses and corresponding to the manufacturer’s recommended human dosage of Mildronate^®^ when converted using the dose translation guide, also ensures comparability with our previous studies demonstrating the protective effects of meldonium (Liepinsh et al., 2009; Nair and Jacob, 2016; Đurašević et al., 2019; Berlato and Bairros, 2020). The 4-week pretreatment duration was based on the meldonium-induced reduction of L-carnitine plateauing in rats at that point, with similar findings in human volunteers, and aligns with the manufacturer’s recommendation for the treatment period with Mildronate^®^ in ischemic conditions (Liepinsh et al., 2009; 2011; Berlato and Bairros, 2020).

### 2.3 FIP-induced sepsis

**FIGURE 1:**
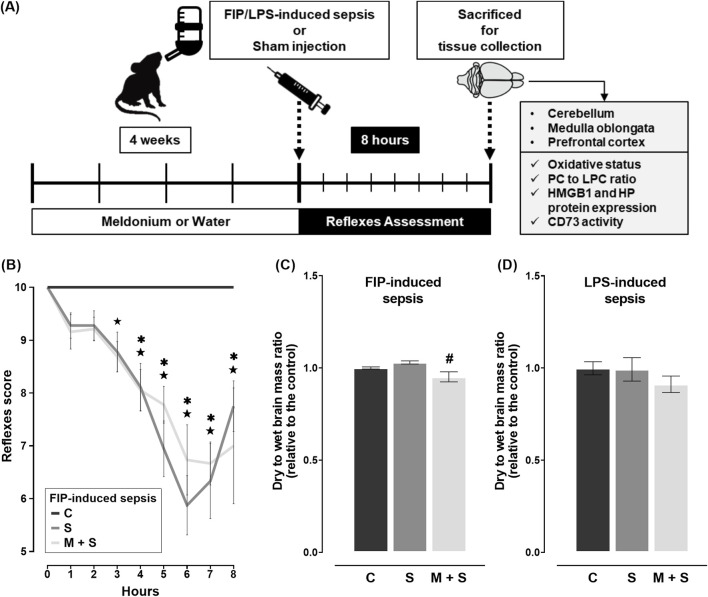
Effect of meldonium pretreatment on reflexes score and dry to wet brain mass ratio in septic rats. **(A)** The same study design was applied in both experimental models of sepsis. **(B)** In FIP-induced sepsis, M + S group showed an earlier and more sustained decline in reflexes score compared to the S group, with both groups demonstrating a slight increase in reflexes score towards the end of the observation period. **(C)** Dry to wet brain mass ratio suggested brain water accumulation in meldonium-pretreated FIP-septic rats, **(D)** while a similar trend was observed in LPS-induced sepsis, but it was not significant. All values are presented as mean ± SEM. *, ★ and # indicate P < 0.05 (*C vs. S; ★ M + S vs. C; #M + S vs. S), as determined by one-way ANOVA with Tukey’s or Kruskal–Wallis with Dunn’s *post hoc* test, depending on data distribution. Abbreviations: FIP, intraperitoneal faecal injection; LPS, lipopolysaccharide; PC, phosphatidylcholine; LPC, lysophosphatidylcholine; CD73, cluster of differentiation 73; HMGB1, high mobility group box 1; HP, haptoglobin; C, sham control; S, sepsis; M + S, meldonium + sepsis.

Nine-week-old male Sprague-Dawley rats weighing 329.91 ± 7.98 g were randomly divided into three experimental groups. The experimental groups were as follows: a sham control group (C) that received only tap water followed by a saline injection (n = 8), a sepsis group (S) that received tap water before sepsis induction (n = 14), and an M + S group (M + S) that received tap water with meldonium before sepsis induction (n = 16). To induce abdominal sepsis, rats were given a single faecal intraperitoneal (i.p.) injection (FIP) (Figure 1A). On the day before sepsis induction, the faeces of control animals were collected from the cage bedding, homogenized in saline solution, and mixed overnight at 4°C to reach a final concentration of 0.5 g/mL. The resulting suspension was filtered twice through quadruple gauze and administered at a dose of 5 g faeces per kg of body mass by a single i.p. with a 25G syringe. To control for bacterial load variability, all rats in the S and M + S groups received the same faecal solution, prepared in a single batch, and the solution was gently shaken before each injection to ensure its uniformity. The rats in the C group were injected i.p. with 10 mL of saline per kg of body mass.

### 2.4 LPS-induced sepsis

Nine-week-old male Sprague-Dawley rats, weighing 240.17 ± 3.81 g, were randomly divided into three experimental groups (n = 8 each), following the same protocol as the FIP-induced sepsis model (Figure 1A). Rats had *ad libitum* access to tap water, with or without meldonium, for 4 weeks before sepsis was induced by a single i. p. injection of lipopolysaccharide (LPS) solution. On the day of sepsis induction, LPS was dissolved in saline to a final concentration of 5 mg/mL. The LPS solution was administered via a single i. p. injection using a 25G syringe at a dose of 5 mg per kg of body mass, a dose reported as effective for inducing SAE (Xin et al., 2022; Dumbuya et al., 2023). The rats in the C group received a single i. p. injection of 1 mL saline solution per kg of body mass.

### 2.5 Animal monitoring and scoring

After the injections, the rats were closely monitored every hour during the 8-h post-sepsis induction period. The presence of pinna reflex, corneal reflex, righting reflex, tail flexion and escape reflex was assessed as described in the literature (Haberham et al., 1999; Kafa et al., 2010). Each of the five reflexes examined was scored as follows: 0 for an absent reflex, 1 for a weak reflex, and 2 for a normal reflex.

### 2.6 Tissue collection and sample preparation

Since the mortality rate in FIP-septic rats pretreated with meldonium increases dramatically from the 8^th^ hour after sepsis induction (Đurašević et al., 2021), to ensure a sufficient group size for subsequent analysis, rats were euthanized at this critical time point for tissue collection. The same endpoints were applied to LPS-induced sepsis to ensure comparability between the two models, although the LPS model is less invasive in terms of observed mortality (Đurašević et al., 2022).

Upon rapid decapitation using a stainless steel guillotine, the brain was quickly removed, and the cerebellum, medulla oblongata, and prefrontal cortex were immediately dissected on ice. Each brain region was then carefully divided into separate sections designated for different analyses described later, ensuring that freeze-thaw cycles were avoided. All samples were snap-frozen using liquid nitrogen and stored separately at −80°C. No samples were collected from rats that did not survive up to 8 h post-injection. A slightly modified previously described protocol was used to prepare the crude membrane fraction (P2) (Stanojlović et al., 2014). Briefly, individual brain structures were homogenized in ice-cold sucrose/Tris-HCl buffer (1:10 mass/volume ratio) using the IKA T 10 Basic Ultra Turrax Homogenizer (IKA^®^-Werke GmbH &Co. KG, Germany) and then centrifuged at 1,000 × g for 10 min at 4°C (Microcentrifuge 5417R, Eppendorf, Germany) to remove unbroken tissue fragments, intact cells, large debris and nuclei and to obtain post-nuclear supernatant. The supernatants were transferred to new tubes and kept on ice, while the remaining pellets containing unhomogenized fragments and nuclei were detached from the tube walls by careful pipetting, resuspended in half of the initial volume of the homogenization buffer, and centrifuged under the same conditions. The resulting supernatants were gathered and centrifuged at 10,000 × g for 20 min at 4°C. After discarding the resulting supernatants, the pellets that contain synaptosomes (nerve terminals), mitochondria, and a proportion of membrane fragments originating from lysed synaptosomes, neuronal, and glial cells, were gently resuspended in ice-cold 5 mM Tris-HCl buffer (pH 7.4). This fraction is referred to as an unpurified synaptosomal or crude membrane fraction (Gray and Whittaker, 1962). The protein concentration was determined according to the modified Lowry method (Markwell et al., 1978). All samples were aliquoted and stored at −80°C until further analysis.

Following dissection of brain regions of interest, the remaining brain tissue was swiftly weighed on an analytical balance to determine the wet mass. Each sample was placed on a piece of aluminum foil and dried in an oven at 180°C until it reached a constant mass, which represents the dry mass. The ratio of dry mass to wet brain mass was calculated and normalized to the C group.

### 2.7 Measurement of tissue oxidative stress indicators

Prooxidant-antioxidant balance (PAB) and advanced oxidation protein products (AOPP) were determined spectrophotometrically as previously described (Todorović et al., 2024).

To estimate concentration of end lipid peroxidation products (LPO) a modified method of Gérard-Monnier was used (Gérard-Monnier et al., 1998). Briefly, methanesulfonic acid was added to the mixtures of sample/standard solution, 1-methyl-2-phenylindole, and ferric chloride in acetonitrile/methanol (3:1 v/v), and the mixtures were vortexed briefly. Following incubation for 1 h at 45°C in dark and centrifugation at 13,000 × g for 15 min at 4°C (Microcentrifuge 5417R, Eppendorf, Germany), the obtained supernatants were transferred to the wells of a microtiter plate, and the absorbance was measured at 586 nm using the Sunrise™ Microplate Reader (Tecan Group Ltd, Switzerland). The standard curve was generated with dilutions of 1,1,3,3-tetramethoxypropane, and sample concentrations were calculated in µM.

### 2.8 Tissue analysis of phosphatidylcholine (PC) to lysophosphatidylcholine (LPC) intensity ratio

Upon total lipid extraction with Folch procedure (Folch et al., 1957), samples were resuspended in 0.5 M DHB (2,5-dihydroxybenzoic acid) matrix solution, placed on the stainless-steel target plate, and allowed to dry to obtain spectra by Voyager DE-Pro MALDI-TOF Mass Spectrometer (AB Sciex, United States) using nitrogen laser (337 nm), under reflector mode and “delayed extraction” conditions (with a delay time of approximately 100 ns) (Todorović et al., 2024). The spectra were processed using Data Explorer software version 4.9 (Applied Biosystems, United states). The most intense peaks of PC and LPC were used to calculate their intensity ratio for each experimental group.

### 2.9 Western blot analysis

Equal amounts of proteins (30 μg) were separated by 8%-15% SDS-PAGE and transferred to a polyvinylidene fluoride (PVDF) membrane (Millipore Sigma, United states). To confirm successful protein transfer, the membranes were stained with Ponceau S staining solution. To block non-specific binding of the primary antibody, PVDF membranes were incubated for 90 min at room temperature in 5% non-fat condensed milk (sc-2324, Santa Cruz Biotechnology, United states) dissolved in TBST buffer (0.2% Tween 20, 50 mM Tris-HCl, pH 7.4, 150 mM NaCl). The membranes were then incubated overnight at 4°C with antibodies against high mobility group box 1 (HMGB1; 1:1,000, ab18256, Abcam, UK) and haptoglobin (1:500, ab256454, Abcam, UK). The membranes were then incubated for 1 h at room temperature with horseradish peroxidase-conjugated secondary antibody (1:30,000, ab6721, Abcam, UK). The immunoreactive bands were detected and recorded using the ChemiDoc-It gel/blot imager (UVP, Germany) after incubating the membranes with the chemiluminescent reagent (Clarity Western ECL, BioRad, United states) for 5 min. To re-probe membranes against the loading control, the secondary antibody was stripped following the Abcam mild stripping protocol and the membranes were then incubated for 1 h at room temperature with a horseradish peroxidase-conjugated anti-β-actin antibody (1:30,000, ab49900, Abcam, UK). Band quantification was performed using ImageJ software.

### 2.10 Measurement of ecto-5′-nucleotidase (CD73) activity

The ecto-5′-nucleotidase (CD73) activity was determined using the malachite green assay as previously reported (Drakulić et al., 2015).

### 2.11 Statistical analysis

Statistical analysis was performed using GraphPad Prism version 8.4.3 (GraphPad Software, Inc., United states). Outliers were excluded from the datasets using the Robust Regression and Outlier Removal (ROUT) test with Q value set to 1%. Datasets that passed the Shapiro-Wilk normality test were subjected to one-way ANOVA analysis followed by *post hoc* analysis using Tukey’s multiple comparisons test. The Kruskal–Wallis test was employed as a non-parametric alternative when the assumptions of normality were violated, and Dunn’s multiple comparisons test was subsequently used for the *post hoc* analysis. To avoid redundancy, only P-values for *post hoc* analyses are reported in the manuscript, along with Z-values for Dunn’s test and degrees of freedom (DF) for Tukey’s test. The threshold for statistical significance was set at P < 0.05. At the endpoints, groups in LPS sepsis had a sample size of n = 8, whereas in FIP sepsis, due to mortality, the sample size was 8, 12, and 8 in the C, S, and M + S groups, respectively. All data are expressed as mean ± standard error of the mean (SEM) and presented graphically relative to the control, unless otherwise stated.

## 3 Results

### 3.1 Effect of meldonium pretreatment on reflexes score and dry to wet brain mass ratio in septic rats

At the end of the 3^rd^ hour post-FIP injection, only the M + S group showed a statistically significant decrease (Z = 2.721, P = 0.0195) in reflexes score compared to the C group (Figure 1B). In the 4^th^ hour post-injection, this decrease became significant in both the S (Z = 2.971, P = 0.0089) and M + S groups (Z = 3.236, P = 0.0036) relative to the C group (Figure 1B). From the 6^th^ hour towards the end of the observation period, both S and M + S groups showed a slight increase in reflexes score with the increase being relatively slower in the M + S group (Figure 1B). No decline in reflexes score was observed in rats with LPS-induced sepsis; hence no graph is included. The dry to wet brain mass ratio of meldonium-pretreated FIP-septic rats was significantly lower (Z = 2.835, P = 0.0137) compared to the sepsis-only group (Figure 1C). A similar but statistically insignificant trend was observed in the M + S group in the LPS model (Figure 1D).

### 3.2 Effect of meldonium pretreatment on oxidative stress biomarkers in the cerebellum, medulla oblongata, and prefrontal cortex of rats with FIP-induced sepsis

**FIGURE 2:**
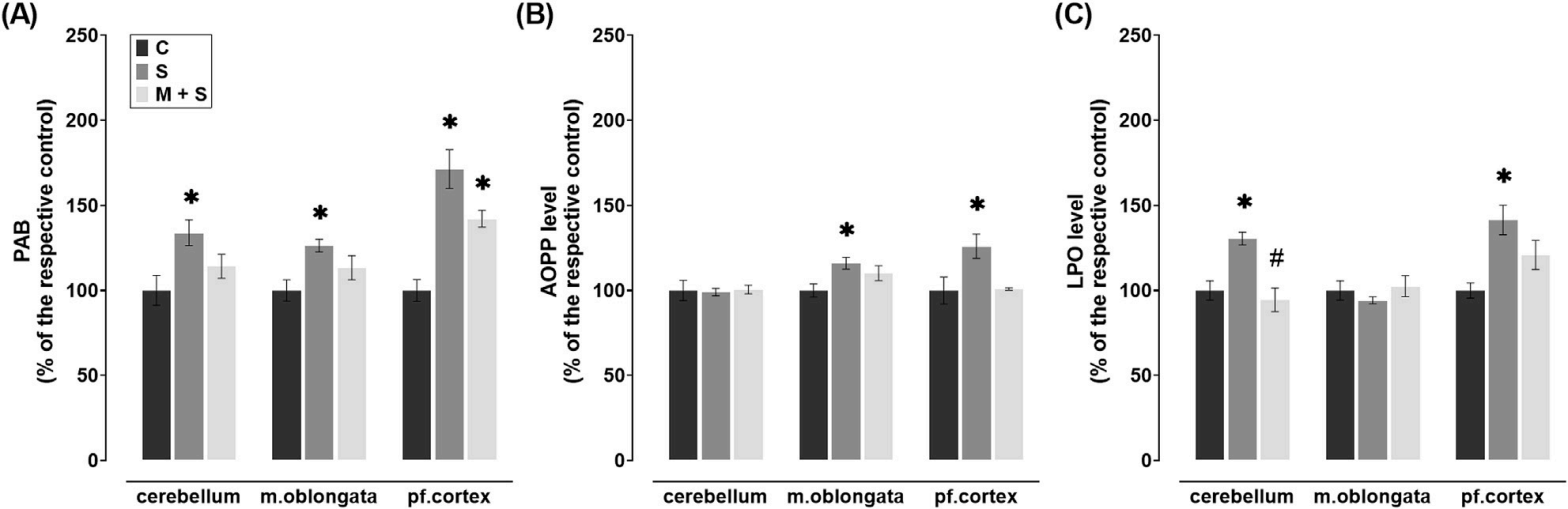
Effect of meldonium pretreatment on oxidative stress biomarkers in cerebellum, medulla oblongata, and prefrontal cortex of rats with FIP-induced sepsis. Meldonium pretreatment prevented increases in **(A)** PAB, **(B)** AOPP, and **(C)** LPO levels observed in different brain regions following FIP-induced sepsis, in a region-specific manner. All values are presented as mean ± SEM. * and # indicate P < 0.05 (* vs. sham control; # vs. sepsis), as determined by one-way ANOVA with Tukey’s or Kruskal–Wallis with Dunn’s *post hoc* test, depending on data distribution. Abbreviations: FIP, intraperitoneal faecal injection; PAB, prooxidant-antioxidant balance; AOPP, advanced oxidation protein products; LPO, lipid peroxidation; m. oblongata, medulla oblongata; pf., prefrontal; C, sham control; S, sepsis; M + S, meldonium + sepsis.

PAB levels were significantly elevated in all three brain regions of rats in the S group (cerebellum: Z = 2.92; P = 0.0103; medulla oblongata: Z = 2.85; P = 0.013; prefrontal cortex: DF = 20, P < 0.0001) in comparison to the C group (Figure 2A). However, in the cerebellum and medulla oblongata of M + S group they were similar to the control values, while in the prefrontal cortex, they were increased (DF = 20, P = 0.0259) compared to the C group (Figure 2A).

FIP sepsis led to a significant increase in AOPP levels in the medulla oblongata (DF = 24, P = 0.0171) and prefrontal cortex (DF = 19, P = 0.0357), whereas no such changes were observed in the rats pretreated with meldonium (Figure 2B). Additionally, similar levels of cerebellar AOPP were observed in all three experimental groups (Figure 2B).

LPO concentration in the cerebellum (DF = 25, P = 0.0009) and prefrontal cortex (DF = 19, P = 0.0055) in the S group increased significantly compared to the C group (Figure 2C). In meldonium-pretreated rats, cerebellar LPO levels were significantly decreased compared to those in FIP-septic rats (DF = 25, P = 0.0001), whereas in the prefrontal cortex no changes were observed (Figure 2C). In medulla oblongata, LPO remained at control values in both S and M + S groups (Figure 2C).

### 3.3 Effect of meldonium pretreatment on oxidative stress biomarkers in the cerebellum, medulla oblongata, and prefrontal cortex of rats with LPS-induced sepsis

**FIGURE 3:**
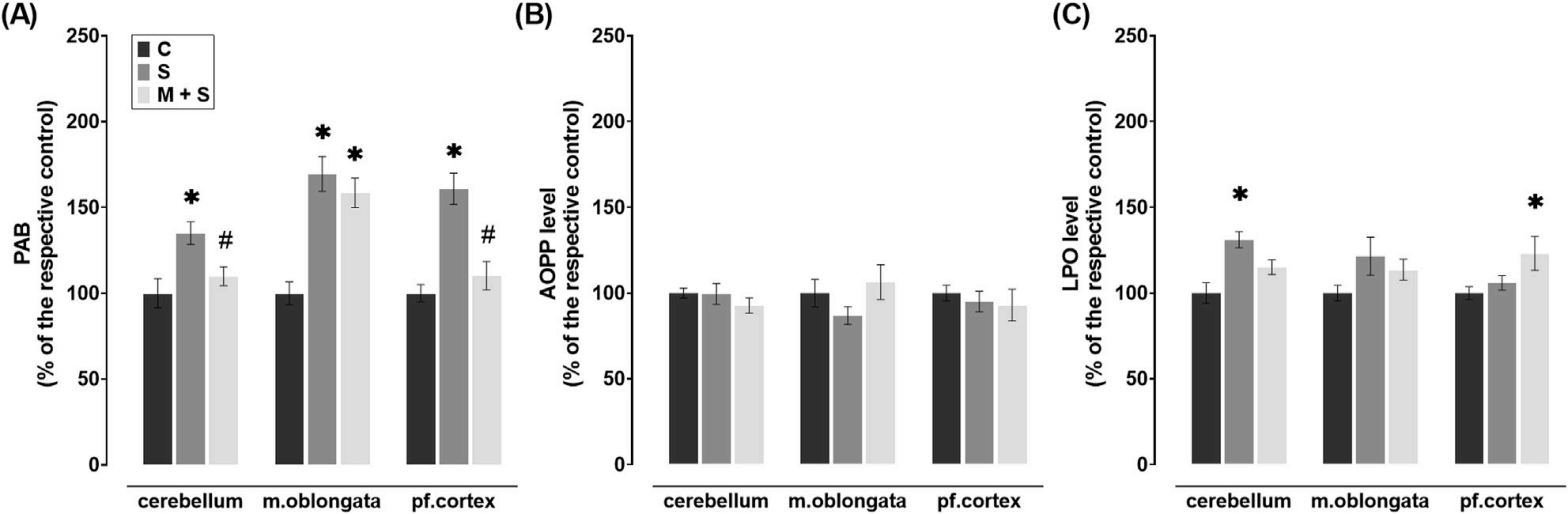
Effect of meldonium pretreatment on oxidative stress biomarkers in cerebellum, medulla oblongata, and prefrontal cortex of rats with LPS-induced sepsis. In LPS-induced sepsis, meldonium maintained **(A)** PAB and **(C)** LPO at sham control levels in a region-specific manner, while **(B)** AOPP levels did not show significant changes across brain regions, with or without meldonium. All values are presented as mean ± SEM. * and # indicate P < 0.05 (* vs. sham control; # vs. sepsis), as determined by one-way ANOVA with Tukey’s or Kruskal–Wallis with Dunn’s *post hoc* test, depending on data distribution. Abbreviations: LPS, lipopolysaccharide; PAB, prooxidant-antioxidant balance; AOPP, advanced oxidation protein products; LPO, lipid peroxidation; m. oblongata, medulla oblongata; pf., prefrontal; C, sham control; S, sepsis; M + S, meldonium + sepsis.

LPS septic rats had a significant increase of PAB in all three brain regions (cerebellum: DF = 21; P = 0.0053; medulla oblongata: DF = 20; P < 0.0001; prefrontal cortex: DF = 20, P < 0.0001) when compared to those in C group (Figure 3A). PAB levels were significantly reduced in comparison to the S group (Figure 3A) in the cerebellum (DF = 21, P = 0.0485) and prefrontal cortex (DF = 20, P = 0.0005) of the M + S group. In parallel, PAB levels in the medulla oblongata of the M + S group were similar to those in the S group and significantly increased compared to the C group (DF = 20, P = 0.0003; Figure 3A).

No significant changes in AOPP levels were observed in any of the brain regions examined after LPS injection, regardless of the pretreatment, when compared to the C group (Figure 3B). In the cerebellum of S group, LPO levels were increased significantly compared to the C group (DF = 20, P = 0.0008), while a similar trend was observed in medulla oblongata, but without statistical significance (Figure 3C). In these brain regions of M + S rats as well as in S rats, LPO levels were comparable to values observed in C group (Figure 3C) while in the prefrontal cortex of the M + S group, this parameter significantly increased (DF = 20, P = 0.0424; Figure 3C).

### 3.4 Effect of meldonium pretreatment on the PC/LPC intensity ratio in the cerebellum, medulla oblongata, and prefrontal cortex of septic rats

**FIGURE 4:**
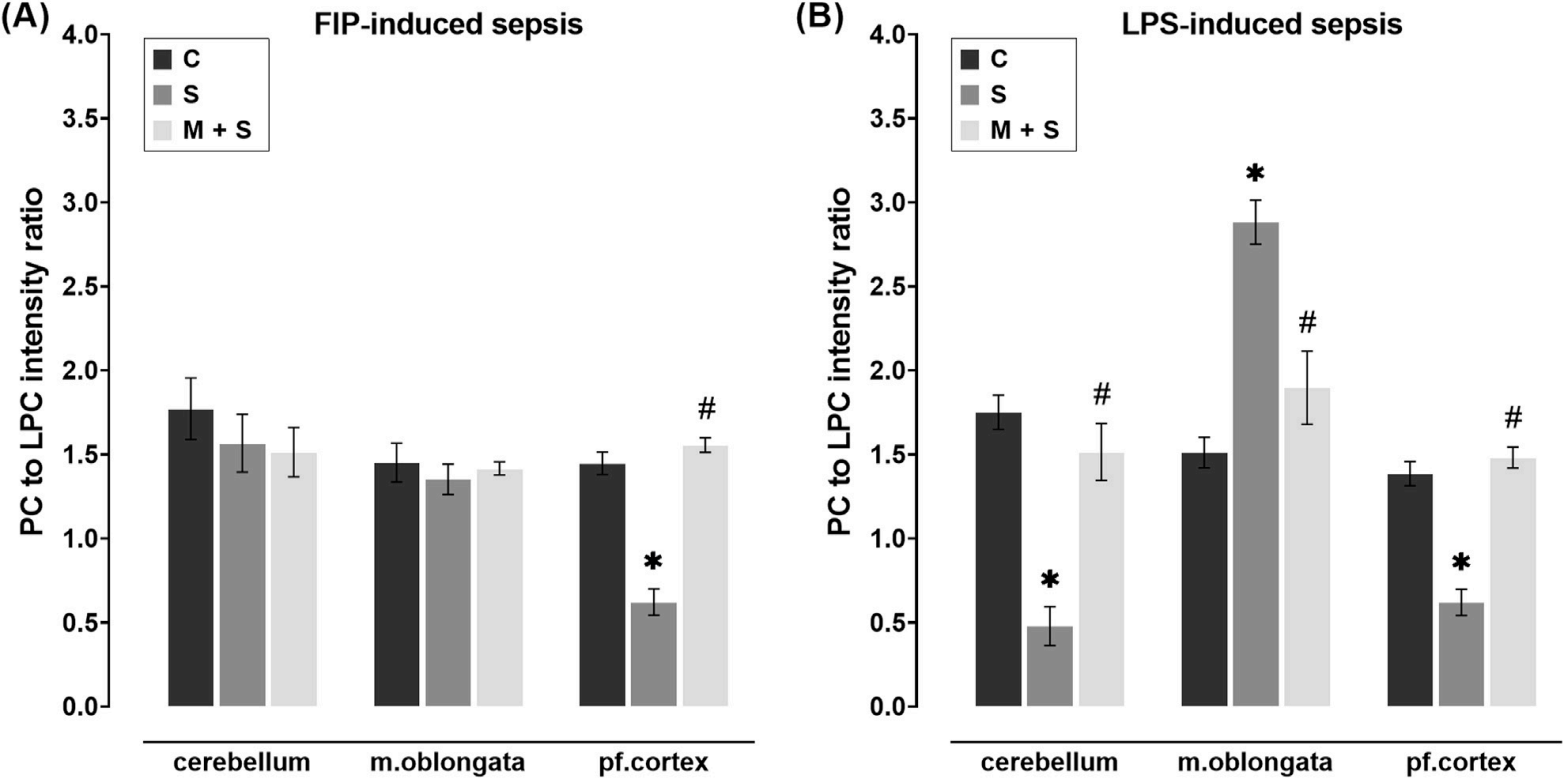
Effect of meldonium pretreatment on PC to LPC intensity ratio in cerebellum, medulla oblongata, and prefrontal cortex of **(A)** FIP-septic rats and **(B)** LPS-septic rats. MALDI-TOF analysis revealed that meldonium maintained the PC/LPC ratio, an indicator of cell membrane integrity, in **(A)** the prefrontal cortex of FIP-septic rats and **(B)** in all three brain regions of LPS-septic rats. All values are presented as mean ± SEM. * and # indicate P < 0.05 (* vs. sham control; # vs. sepsis), as determined by one-way ANOVA with Tukey’s or Kruskal–Wallis with Dunn’s *post hoc* test, depending on data distribution. Abbreviations: FIP, intraperitoneal faecal injection; LPS, lipopolysaccharide; PC, phosphatidylcholine; LPC, lysophosphatidylcholine; m. oblongata, medulla oblongata; pf., prefrontal; C, sham control; S, sepsis; M + S, meldonium + sepsis.

In obtained mass spectra in all experimental groups, the PC and LPC peaks were detected in two regions, 490–550 and 750–830 m/z. In sepsis groups of both experimental models, the PC/LPC intensity ratio in the prefrontal cortex was significantly decreased compared to the C group (both models: DF = 12; P < 0.0001; Figures 4A,B). However, in M + S groups of both experimental models, PC/LPC ratio in the prefrontal cortex remained at control values and was significantly higher in comparison to the respective S group (both models: DF = 12; P < 0.0001; Figures 4A,B). When compared to C rats, in the cerebellum and medulla oblongata of FIP-septic rats, the PC/LPC ratio remained unchanged regardless of the pretreatment (Figure 4A). In LPS rats, however, the PC/LPC ratio was decreased in the cerebellum (DF = 11, P = 0.0001) but increased in the medulla oblongata (DF = 12, P = 0.0001) compared to C animals (Figure 4B). In these two structures in meldonium-pretreated LPS-septic rats, PC/LPC ratio was maintained at levels observed in C group (Figure 4B).

### 3.5 Effect of meldonium pretreatment on the HMGB1 and haptoglobin content in the cerebellum, medulla oblongata, and prefrontal cortex of rats with FIP-induced sepsis

**FIGURE 5:**
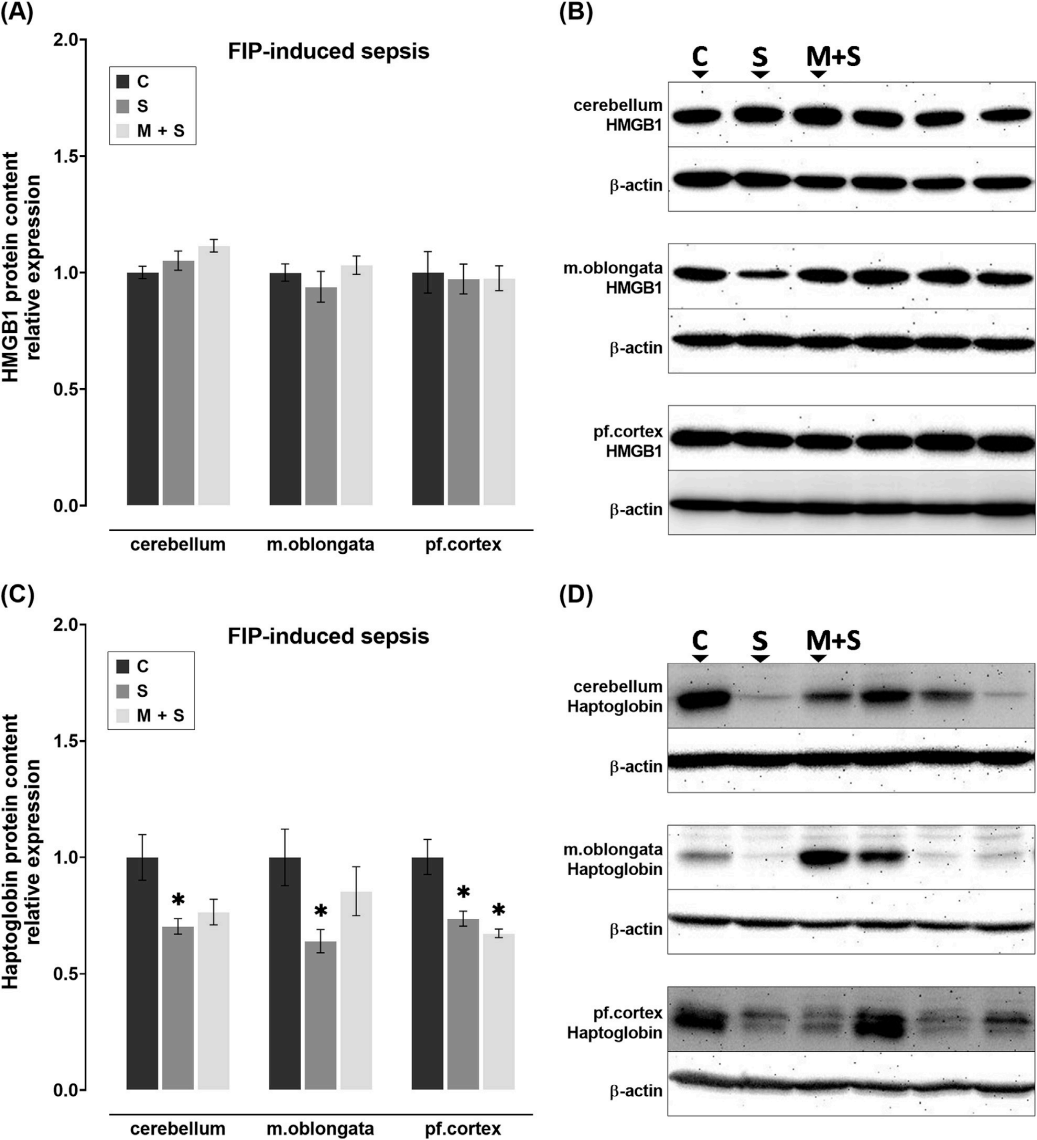
Effect of meldonium pretreatment on HMGB1 and haptoglobin content in cerebellum, medulla oblongata, and prefrontal cortex of rats with FIP-induced sepsis. **(A,B)** Following FIP-induced sepsis, no changes in HMGB1 protein content were observed in either the S or M + S groups, as revealed by Western blot analysis. **(C,D)** Meldonium pretreatment significantly preserved haptoglobin content in the cerebellum and medulla oblongata, indicating a protective effect against FIP sepsis-induced haptoglobin depletion. All values are presented as mean ± SEM. * and # indicate P < 0.05 (* vs. sham control; # vs. sepsis), as determined by one-way ANOVA with Tukey’s or Kruskal–Wallis with Dunn’s *post hoc* test, depending on data distribution. β-actin was used as a loading control. Abbreviations: FIP, intraperitoneal faecal injection; HMGB1, high mobility group box 1; m. oblongata, medulla oblongata; pf., prefrontal; C, sham control; S, sepsis; M + S, meldonium + sepsis.

No statistically significant changes in HMGB1 protein content were observed in any of the brain regions of FIP-septic rats in comparison to the C group (Figures 5A,B). In contrast, haptoglobin protein content was decreased in all three brain regions of rats in the S group compared to the C group (cerebellum: Z = 2.739, P = 0.0185; medulla oblongata: DF = 21; P = 0.0297; prefrontal cortex: DF = 17, P = 0.0029; Figures 5C,D). Such trend was observed in the M + S group in all three brain regions, but was statistically significant in the prefrontal cortex only (DF = 17, P = 0.0011; Figures 5C,D).

### 3.6 Effect of meldonium pretreatment on the HMGB1 and haptoglobin content in the cerebellum, medulla oblongata, and prefrontal cortex of rats with LPS-induced sepsis

**FIGURE 6:**
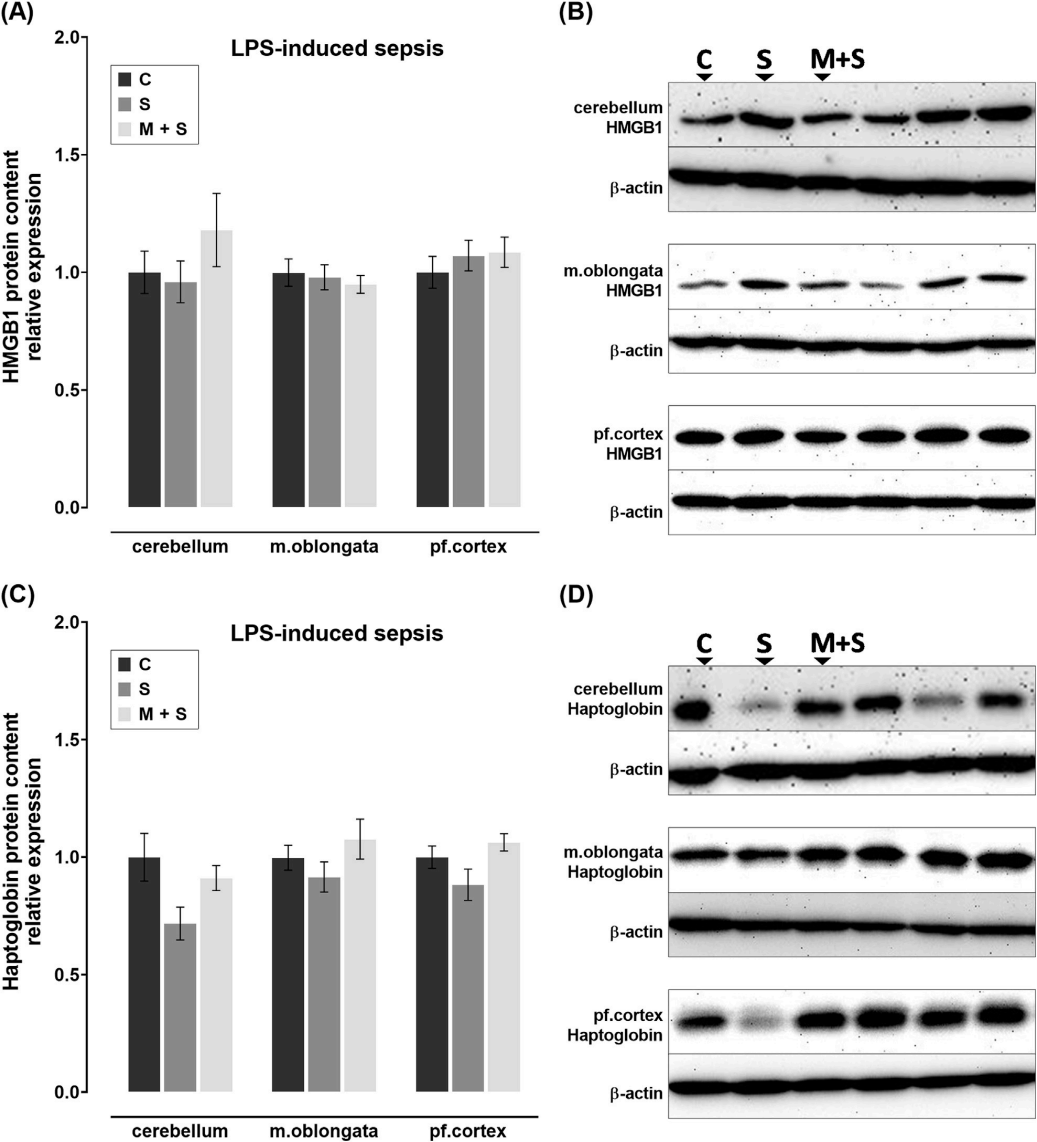
Effect of meldonium pretreatment on HMGB1 and haptoglobin content in cerebellum, medulla oblongata, and prefrontal cortex of rats with LPS-induced sepsis. Following LPS-induced sepsis, no changes in **(A,B)** HMGB1 or **(C,D)** haptoglobin protein content were observed in any of the investigated brain regions, with or without meldonium, as revealed by Western blot analysis. All values are presented as mean ± SEM. * and # indicate P < 0.05 (* vs. sham control; # vs. sepsis), as determined by one-way ANOVA with Tukey’s or Kruskal–Wallis with Dunn’s *post hoc* test, depending on data distribution. β-actin was used as a loading control. Abbreviations: LPS, lipopolysaccharide; HMGB1, high mobility group box 1; m. oblongata, medulla oblongata; pf., prefrontal; C, sham control; S, sepsis; M + S, meldonium + sepsis.

No statistically significant changes in HMGB1 protein levels were observed in rats with LPS-induced sepsis, regardless of pretreatment (Figures 6A,B). Although not statistically significant, compared to C group, haptoglobin levels appeared to be downregulated in S group across all three brain structures; while they remained at control levels in M + S group (Figures 6C,D).

### 3.7 Effect of meldonium pretreatment on CD73 activity in the cerebellum, medulla oblongata, and prefrontal cortex of septic rats

**FIGURE 7:**
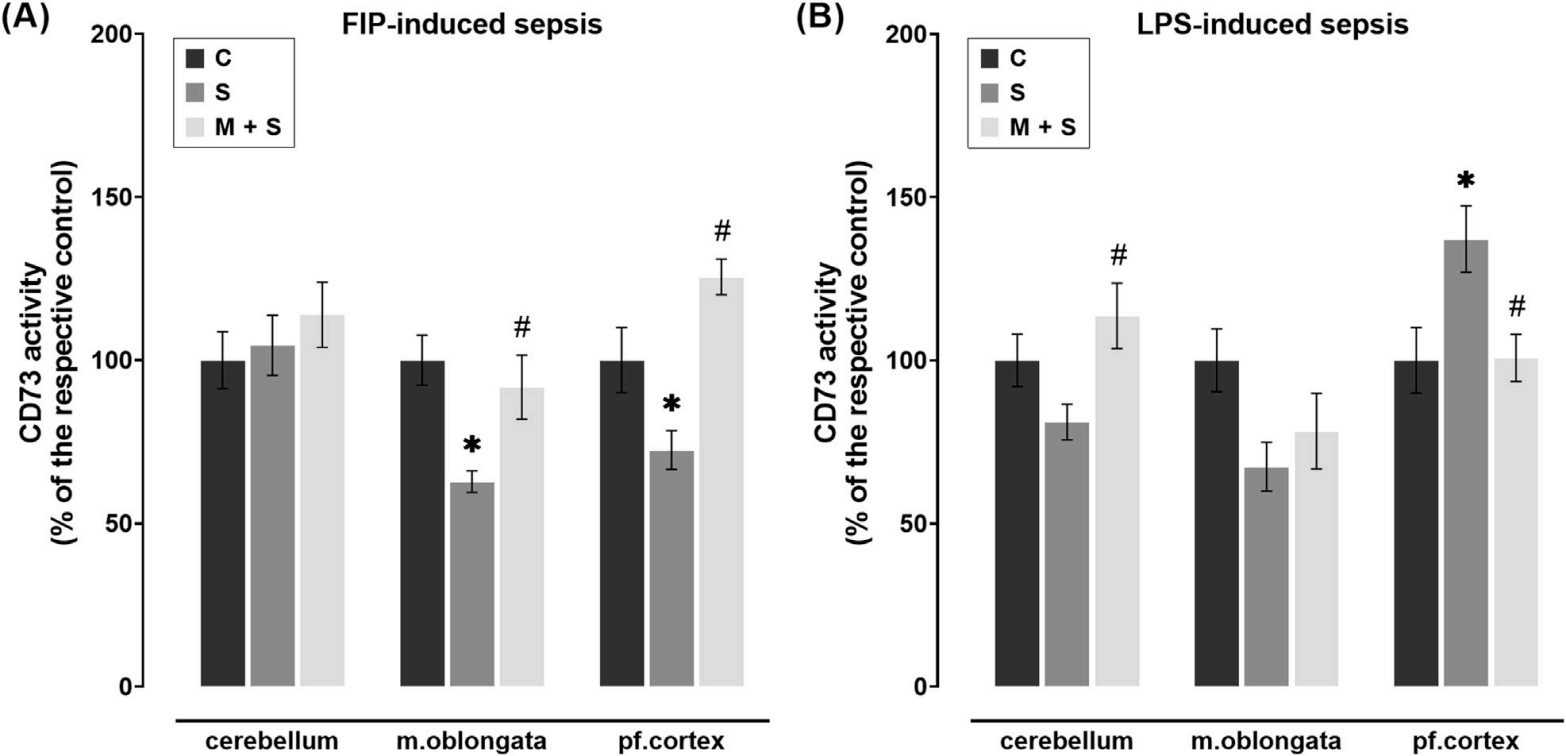
Effect of meldonium pretreatment on CD73 activity in cerebellum, medulla oblongata, and prefrontal cortex of **(A)** FIP-septic rats and **(B)** LPS-septic rats. Enzyme assay revealed that pretreatment with meldonium preserved CD73 activity **(A)** in the medulla oblongata and prefrontal cortex of FIP-septic rats, as well as **(B)** in the cerebellum and prefrontal cortex of LPS-septic rats, suggesting a protective effect that is dependent on both the brain region and the sepsis model investigated. All values are presented as mean ± SEM. * and # indicate P < 0.05 (* vs. sham control; # vs. sepsis), as determined by one-way ANOVA with Tukey’s or Kruskal–Wallis with Dunn’s *post hoc* test, depending on data distribution. Abbreviations: FIP, intraperitoneal faecal injection; LPS, lipopolysaccharide; CD73, cluster of differentiation 73; m. oblongata, medulla oblongata; pf., prefrontal; C, sham control; S, sepsis; M + S, meldonium + sepsis.

The AMP hydrolysis rate, as an indicator of CD73 activity, was markedly reduced in the medulla oblongata (DF = 25, P = 0.0012) and prefrontal cortex (DF = 21, P = 0.0309) of the S group in comparison to the C group (Figure 7A). In these brain regions of FIP-septic rats pretreated with meldonium, CD73 activity was increased significantly compared to the S group (medulla oblongata: DF = 25; P = 0.008; prefrontal cortex: DF = 21, P = 0.0001), whereas its cerebellar activity was similar between experimental groups (Figure 7A).

No statistically significant changes in CD73 activity were observed in the medulla oblongata of either meldonium-pretreated or non-pretreated LPS-septic rats in comparison to C group (Figure 7B). In two other regions, opposite patterns of CD73 activity were observed, with a higher AMP hydrolysis rate in the cerebellum (DF = 18, P = 0.0214) but a lower rate in the prefrontal cortex (DF = 19, P = 0.0365) of M + S rats compared to the S group (Figure 7B).

## 4 Discussion

Meldonium, marketed under the brand name Mildronate^®^ and prescribed for ischemic conditions of the heart and brain, exerts its effects by inhibiting gamma-butyrobetaine hydroxylase, leading to reduced tissue levels of L-carnitine (Dambrova et al., 2016). This causes a shift in energy metabolism from FAs oxidation to glucose utilization, which is of particular interest as downregulation of lipid metabolism has been shown to reverse or slow the progression of certain central nervous system disorders (Trabjerg et al., 2020). Although meldonium has been recognized for its neuroprotective properties (Sjakste et al., 2005; Pupure et al., 2010a; Isajevs et al., 2011; Beitnere et al., 2014; Demir et al., 2019; Ozaydin et al., 2023), its effects in the context of SAE, one of the most common sepsis-associated complications (Catarina et al., 2021), remain unknown. To clarify the potential effects of meldonium in the SAE, we examined markers of brain injury in rats receiving meldonium via drinking water for 4 weeks prior to induction of abdominal sepsis in two different models.

In SAE, impaired neurobehavior manifests early and is usually evaluated by reflex testing both in intensive care unit settings and in animal models (Pan et al., 2022; Qin et al., 2023). In FIP septic rats, reflexes score began to deteriorate early regardless of the pretreatment, but from the 4^th^ hour onwards, this decline was milder in the M + S group. This finding is consistent with the results of another invasive model, in which meldonium improved the righting reflex in rats after a single i.p. following ethanol-induced neurotoxicity (Zvejniece et al., 2010). However, in both sepsis groups, we observed a slight improvement in reflexes after the 6^th^ and 7^th^ hours following FIP injection. The observed improvement likely reflects contributions from animals that exhibited higher initial scores and a more favourable response to the septic conditions, as those with the lowest initial scores had already succumbed to sepsis. Thus, this apparent improvement reflects the behaviour of rats better able to withstand the sepsis-induced insult and is consistent with previous studies that differentiated individuals based on the severity of sepsis they developed (Horiguchi et al., 2018; Zhang H. et al., 2022).

Sepsis-associated pathophysiological changes leading to impaired neurobehavior, include brain perivascular edema due to the impairment of BBB by both pathogens and systemically elevated pro-inflammatory markers (Kuperberg and Wadgaonkar, 2017). While we did not observe changes in the dry to wet brain mass ratio in sepsis alone, it appears that sepsis preceded by meldonium pretreatment led to water accumulation in the brain, as there was a trend towards a slight decrease in this parameter in both sepsis models, but was statistically significant only in the FIP model. Insufficient perfusion of the brain due to blood vessels being compressed by edema leads to an inadequate supply of oxygen and nutrients, which rapidly manifests as a poor mental state and lowered reflexes score, and can eventually lead to coma or death (Pan et al., 2022; Sekino et al., 2022). We propose that the previously observed high mortality rate in septic rats pretreated with meldonium (Đurašević et al., 2021; 2022) may partly stem from the development of brain edema. Meldonium’s carnitine-lowering effects (Dambrova et al., 2016; Berlato and Bairros, 2020; Shaforostova et al., 2021) induce carnitine deficiency, thereby possibly impairing astrocyte function (Trabjerg et al., 2020; Shaforostova et al., 2021; Szrok-Jurga et al., 2023) and potentially predisposing rats to BBB damage and brain edema when they are subjected to sepsis.

Together with BBB impairment, the complex pathophysiology of SAE is marked by an imbalance between generation and scavenging of reactive oxygen species (ROS) and reactive nitrogen species (RNS), triggering reactions that damage macromolecules, thereby compromising cellular structure and function (Sekino et al., 2022). One of the key indicators of cell membrane integrity is the PC/LPC ratio, as PC is essential for membrane stability, while LPC is generated from it during damage and can act as a bioactive lipid, activating pro-inflammatory pathways that further destabilize membranes (Liu et al., 2020). Therefore, a decrease in PC relative to LPC is considered indicative of cell membrane degradation. To better understand the mechanisms underlying the susceptibility of meldonium-pretreated rats to SAE, we measured levels of PAB, AOPP, LPO, and PC/LPC ratios in different brain regions to assess tissue oxidative status and the extent of protein and lipid damage caused by oxidative stress. Given the overwhelming inflammation in response to infection in sepsis, the protein content of the inflammatory markers HMGB1 and haptoglobin was evaluated. We also measured CD73 activity due to its role as a rate-limiting enzyme in converting extracellular ATP (eATP), released by damaged cells, into adenosine, which has been shown to increase in both plasma and brain following i.p. LPS injection (Guo et al., 2024).

Motor control and coordination can be severely impaired in SAE, leading to deficits in balance, posture, and fine motor skills (Zhao et al., 2024). Given the critical role of cerebellum in regulating these processes, we examined the effects of meldonium pretreatment in this brain region of septic rats. In the cerebellum in both sepsis models, oxidative stress was observed, as indicated by increased levels of PAB and LPO. The observed phenomena appear to be localized and primarily target lipids, while sparing proteins from damage, as cerebellar levels of AOPP, and HMGB1, along with CD73 activities, remained unchanged in both models. This was further supported in LPS sepsis by the reduced cerebellar PC/LPC ratio, suggesting that lipids within membranes sustained structural damage, possibly due to increased LPO. A 4-week meldonium pretreatment appears to be sufficient to modulate FA metabolism selectively and inhibit the accumulation of toxic FA derivatives, consistent with the documented neuroprotective effects of meldonium (Sjakste et al., 2005; Pupure et al., 2010a; Isajevs et al., 2011; Beitnere et al., 2014; Demir et al., 2019; Ozaydin et al., 2023). This is also supported by a decreased LPO in the cerebellum of meldonium-pretreated FIP septic rats and an elevated PC/LPC ratio in the same brain region of meldonium-pretreated LPS septic rats.

Medulla oblongata, with its nuclei regulating autonomic functions including heart rate and blood pressure control, may be particularly prone to sepsis-induced damage due to its proximity to the area postrema (Heming et al., 2017). The presented results suggest that sepsis-induced oxidative stress affects protein and lipid compartments in this brain region differently than in the cerebellum. In rats with FIP sepsis, elevated PAB and AOPP levels, along with reduced haptoglobin expression and CD73 activity, indicate significant protein damage in this region. However, oxidative lipid damage appears well controlled, as evidenced by stable LPO levels and PC/LPC ratio. Pretreatment with meldonium might help to prevent the impairment of vital functions regulated by this brain region, as our results showed that all markers of oxidative stress and inflammation in the medulla oblongata of meldonium-pretreated FIP septic rats were maintained at control levels. In the medulla oblongata of LPS septic rats, the observed increase in the PC/LPC ratio, in addition to stable LPO levels, might be associated with certain LPO-independent adjustments in lipid metabolism. Meldonium pretreatment appears to specifically target these LPO-independent processes, as PC/LPC ratio in the medulla oblongata of LPS septic rats was significantly decreased compared to sepsis only.

The prefrontal cortex, crucial for higher cognitive functions, suffers significant impairment due to SAE, leading to severe cognitive deficits and behavioural changes (Heming et al., 2017). Sepsis-induced damage in this region following FIP sepsis was extensive, as almost all examined markers were disrupted. The observed increase in oxidative stress markers, along with a reduced PC/LPC ratio, haptoglobin levels, and CD73 activity, indicated significant oxidative damage affecting both lipids and proteins. The extent of injury appeared to be lesser in LPS septic rats, in which LPO, AOPP, and haptoglobin levels remained unchanged. In both models, pretreatment with meldonium helped return the PC/LPC ratio to control values, indicating that meldonium selectively affects lipid metabolism in this structure as well. However, the effects of meldonium pretreatment appear to be less pronounced when followed by FIP sepsis, as haptoglobin levels were similar in both septic groups.

Certain model-specific effects of meldonium pretreatment could be attributed to the complex nature of FIP-induced polymicrobial sepsis, whereby it may prevent some aspects of tissue damage, albeit to a limited extent. The FIP model is considered more invasive, as a variety of microbial components and live bacteria are introduced into the peritoneal cavity mimicking clinical cases of peritonitis (Seemann et al., 2017; Tenhunen et al., 2020). In contrast, the LPS model, exposes animals to a single component from the outer cell wall of Gram-negative bacteria, making it a single endotoxin-driven and therefore more controlled form of sepsis (Seemann et al., 2017; Tenhunen et al., 2020). Thus, the FIP model triggers a broader and potentially clinically more relevant inflammation, while the LPS model induces a more straightforward inflammatory response due to being triggered by a single endotoxin (Seemann et al., 2017). Therefore, the LPS model is considered more suitable for studying the molecular mechanisms of sepsis and offers greater reproducibility due to its well-defined induction process, while polymicrobial models (e.g., cecal ligation and puncture (CLP) and FIP) more accurately reflect the physiological changes observed in septic patients (Zhang et al., 2017). The model-specific variations we observed in the context of SAE contribute to the ongoing debate about the suitability of different models for studying various aspects of sepsis (Remick et al., 2000; Zhang et al., 2017). The greater severity of the FIP model was perhaps most evident in the significant reduction in haptoglobin protein content across all three brain regions. This reduction is likely due to the fact that haptoglobin binds and degrades cell-free haemoglobin, and its deficiency further compromises tissue defence against oxidative damage (Spagnuolo et al., 2015). Our findings also emphasize the importance of analysing individual brain regions under septic conditions, rather than the whole brain. Namely, we observed region-specific increases in LPO in both FIP- and LPS-induced sepsis. Our findings in LPS sepsis align with reports of an early and persistent LPO increase in the whole brain of LPS-injected mice (Seemann et al., 2017), but this increase was not observed in polymicrobial sepsis models (Remick et al., 2000; Zhang et al., 2017).

Apart from model-specific differences, the present study also revealed a varying trend of meldonium effects in different brain regions. Interestingly, meldonium prevented a decrease in haptoglobin content in the cerebellum and medulla oblongata, but not in the prefrontal cortex of FIP-septic rats. The lower efficacy of meldonium in the prefrontal cortex, compared to the cerebellum and medulla oblongata of FIP-septic rats, was also evident in the amount of PAB. This might be attributed to the reported differences in the extent of sepsis-induced injury across brain regions (Kafa et al., 2010; Silverman et al., 2014; Heming et al., 2017) which are associated with higher microglial density in the cortical regions and lower density in the cerebellum and brainstem (Tian et al., 2019). Kim et al. observed that neurons in the substantia nigra are more severely damaged under septic conditions than those in the hippocampus and cortex (Kim et al., 2000). Similarly, in septic mice, IL1b expression was higher in the cortex than in the cerebellum and brainstem (Silverman et al., 2014). Therefore, it is plausible to hypothesize that meldonium’s efficacy in preventing sepsis-induced injury varies across brain regions due to regional differences in the extent of damage caused by sepsis. It is noteworthy that, in contrast to FIP, PAB levels in the medulla oblongata of LPS septic rats remained elevated despite meldonium pretreatment. This could be attributed to the inherently high basal ROS levels, intense mitochondrial and metabolic activity (Anusha-Kiran et al., 2022), and elevated pro-inflammatory cytokine expression within this region during infection (Soung et al., 2022). These factors, along with LPS model generally considered to cause a more rapid, acute inflammatory response (Đurašević et al., 2022), could limit the efficacy of antioxidant interventions like meldonium.

Purinergic signaling plays an important role in the pathophysiology of sepsis-induced brain injury (Ledderose et al., 2016). A well recognized “danger” signal, eATP, increases in response to cellular damage and inflammation during sepsis and septic shock (Di Virgilio et al., 2009; Ledderose et al., 2016). Through P2X7 as a primary receptor, eATP induces microglial cell activation and migration to injury sites, where they form a barrier between healthy and damaged tissue (Di Virgilio et al., 2009; Monif et al., 2009). However, P2X7 activation is also linked to increased ROS production, exacerbating tissue damage, while P2X7 deletion has been shown to reduce oxidative stress in brains of septic mice (Savio et al., 2017). ATP-dependent signaling mediated by the P2X7 receptor is modulated by ectonucleotidases expressed on the surface of microglial cells, which catalyse a series of hydrolysis steps that break down ATP, with CD73 catalysing the final step, converting AMP to adenosine (Zimmermann, 1992; Savio et al., 2017). The role of CD73 in sepsis-related injury has been recognized mainly through its pharmacological inactivation and studies in knockout animals (Reutershan et al., 2009; Haskó et al., 2011). While its activity within the brain has been previously explored in LPS-induced neuroinflammation (Smith et al., 2014; Miron et al., 2023), it has not yet been studied in polymicrobial sepsis models. Perhaps most remarkably in the present study, is that CD73 activity in the prefrontal cortex is decreased in FIP-septic rats, whereas it is increased in LPS-septic rats. Previous studies reported no changes in brain CD73 activity in response to LPS (Smith et al., 2014; Miron et al., 2023), but this discrepancy might stem from methodological differences, as Miron et al. assessed CD73 activity in the rat cerebral cortex 24 h after the i.p. LPS injection, while Smith et al. measured it in the brain continuously perfused with LPS for 14 or 28 days. Our finding is also in line with a recent study showing that peripheral LPS challenge causes a rapid increase of extracellular adenosine in brain (Guo et al., 2024). Altogether, our findings suggest that CD73 is involved in the early phases of sepsis as a neuroprotective factor, owing to the massive production of adenosine, which acts as a modulatory, immunosuppressive, anti-inflammatory, and protective agent (Haskó et al., 2011; Ledderose et al., 2016; Zhang T. et al., 2022). In line with the protective role of CD73-derived adenosine, several studies have indicated that diminished CD73 function may be a contributing factor to severe or persistent infections (Gao et al., 2022; Xiang et al., 2025). In sepsis, where excessive inflammation and cytokine release occur, increased CD73 activity may reflect the body’s attempt to counteract immune overactivation and limit tissue damage. Similar to the previously shown correlation between plasma adenosine levels and septic shock incidence (Gao et al., 2022), we hypothesized that the changes in CD73 activity we observed correlate with the severity or progression of neuroinflammation in SAE.

## 5 Conclusion

Four weeks of meldonium pretreatment demonstrated neuroprotective effects in septic rats, particularly in the cerebellum and medulla oblongata, with the extent of these effects depending on the sepsis model (FIP vs. LPS). Across both models, however, the benefits of meldonium pretreatment were generally less pronounced in the prefrontal cortex. Our findings highlight the importance of considering both the sepsis model and regional brain variations when modelling SAE and exploring new therapeutic interventions.

### 5.1 Study limitations and recommendations

While our findings offer important novel insights, several limitations should be considered and addressed in future studies. The short observation window (up to 8 h post-sepsis induction), due to the high mortality rate in the FIP model, limited our ability to assess the long-term effects of meldonium on the progression of SAE. Additionally, although this is the first study to demonstrate the benefits of meldonium pretreatment in the context of SAE, an in-depth exploration of the underlying molecular mechanisms of its neuroprotective effects remains necessary. This includes further investigation into the brain edema observed in meldonium-pretreated FIP-septic rats. Regional differences in BBB integrity under septic conditions should also be addressed, as these could help explain the observed discrepancies in the direction of changes in some markers across brain regions. Finally, given the lack of sepsis-specific treatments, our study paves the way for exploring the protective potential of other drugs with similar mode of action similar to meldonium (e.g., perhexiline), which could be successfully repurposed for sepsis treatment.

## Data Availability

The raw data supporting the conclusions of this article will be made available by the authors, without undue reservation.
